# Rapid diagnosis of acute pediatric respiratory infections with Point-of-Care and multiplex molecular testing

**DOI:** 10.1007/s15010-025-02553-5

**Published:** 2025-05-26

**Authors:** Jane M. Caldwell, Claudia Mily Espinosa, Ritu Banerjee, Joseph B. Domachowske

**Affiliations:** 1 Medavera, Inc., Springfield, MO 65807 USA; 2 Division of Pediatric Infectious Diseases, University of South Florida, Tampa, FL USA; 3 Division of Pediatric Infectious Diseases, Vanderbilt University Medical Center, Nashville, TN USA; 4 Upstate Medical University, Syracuse, NY USA

**Keywords:** Acute respiratory infection, Molecular diagnostics, Diagnostic methods, Point-of-care, Multiplex testing, Influenza, Respiratory syncytial virus, COVID-19; disease surveillance

## Abstract

Acute infections of the respiratory tract are very common in pediatric patients, with an estimated global incidence of 17.2 billion cases in 2019. Accurate and timely diagnosis and treatment of acute respiratory infections can prevent progression to more serious pathologies, especially in the young, elderly, immunocompromised, and other high-risk groups. Due to the significant increase in the number of multiplex molecular tests available, there are now many diagnostic options which generate results within minutes or hours, many of which can be performed at point-of-care or near-patient rather than being sent out to a centralized laboratory. Rapid molecular single- or multiplex testing conducted at point-of-care or near-patient offers the potential to improve timely and accurate diagnosis, decrease inappropriate antibiotic use, decrease reliance on chest radiographs, improve timely antiviral administration, reduce the length of hospital stay, reduce the number of clinical visits, and, ultimately, improve patient outcomes. Optimal use of user-friendly multiplex molecular panels also has the potential to improve regional and global disease surveillance and to fill gaps that exist in our understanding of the epidemiology of respiratory infections. These potential benefits, however, come with limitations. For example, use of multiplex PCR assays is not always a cost effective approach. Despite their potential, there are clinical and/or laboratory circumstances where their use becomes cost prohibitive. Another recognized limitation of multiplex PCR assays is that the pathogen detected may not be the cause of a patient’s current symptom complex. Such false positive results may occur because the assays are designed to detect pathogen-specific nucleic acid (which may be residual from a prior illness), rather than replication competent pathogens, or because some pathogens can be present without causing symptomatic infection. Further study is needed to determine optimal use of these tests across different patient groups and settings. Incorporating recommendations for best practice use of multiplex molecular assays into clinical guidelines helps offer a framework for their most appropriate use in the diagnosis of pediatric acute respiratory infections.

## Introduction

The use of multiplex molecular testing methods for rapid diagnosis of pediatric infectious diseases continues to expand, and even replace, conventional methods in many instances. Some studies have reported clinical, analytical, and cost benefits with the use of these panels for the detection of respiratory pathogens while other studies have shown no clear benefits. Some medical societies have published clinical guidance documents recommending the use of molecular panels to diagnose and manage certain patient populations [1]. Here we explore advantages and shortcomings of their use and consider clinical utility, cost analysis, insurance coverage, workflow, disease screening, epidemiological surveillance, and patient outcomes.

### Acute respiratory infections: disease burden

Acute infections of the respiratory tract (ARIs) are very common, with an estimated global incidence of 17.2 billion cases in Incidence and morbidity between 1990 and 2019 were highest at younger ages with increased mortality seen among the elderly and children younger than 5 years of age [2]. While some upper respiratory tract infections can be severe, even life-threatening (epiglottitis in particular), many are benign and self-limiting. Complications of ARIs are generally infrequent, but do occur, thereby contributing to the global burden of respiratory infections as a whole [3]. In contrast, lower respiratory tract infections (LRTIs) are a leading cause of hospitalization and mortality worldwide with approximately 500 million cases and 2.5 million deaths occurring globally in 2019 alone [4]. Not unexpectedly, mortality is reported more frequently from countries with fewer resources [4, 5]. Between 1990 and 2019 the overall incidence and mortality from LRTIs has trended downward in the pediatric population, likely reflecting the efforts of targeted vaccination against the most common bacterial causes and decreasing known risk factors for respiratory infections [4]. In the United States, a recent analysis from the Kids’ Inpatient Database (KID), demonstrated that the 3 most common causes of hospitalization among patients under 18 years of age were bronchiolitis, pneumonia and asthma [6]. During the first 2 years of life, it is estimated that, on average, children have between 2 and 8 respiratory infections. The average number of respiratory tract infections each year among those who attend day care is higher, with some children experiencing as many as 14 annually. Younger age is also associated with higher rates of complications from acute respiratory infections [7, 8].

ARI symptoms in children often do not differ between viral and bacterial etiologies and are not considered pathogen specific. Overlap in ARI symptoms leads to a level of uncertainty regarding the underlying microbiologic diagnosis and need for antibiotics [9]. Before molecular assays became widely available in most clinical virology laboratories, diagnostic tools were limited to virus cultures, various virus antigen detection techniques, and serologic assays. Each of these assay types remain available today, but many are no longer used as first-line testing because, in general, molecular techniques have higher sensitivity, faster turn-around times, and a broader repertoire of multiplex testing capacity [10–12]. Performing and interpreting viral culture results requires high-level training, experience and expertise while many multiplex molecular assays can also now be performed at point-of-care or near-patient by personnel with minimal training or experience due to advances in automation [13–15].

### Epidemiology of acute respiratory infections

The incidence of ARIs in children varies by year, geography, and season. The majority of these infections in temperate areas of the world occur during the winter ‘cold and flu’ season, while tropical areas may experience longer seasons or year-round incidence, particularly for respiratory syncytial virus (RSV) and influenza. In the last several years, the timelines for seasonal outbreaks have shifted likely due in part to global travel, changes in climate, and a variety of factors related to the COVID-19 pandemic. Traditionally, surveillance of respiratory viruses has focused largely on patterns of RSV, influenza, and more recently, SARS-CoV-2 activity. Results of passive surveillance are challenging to interpret because not all children and infants with lower respiratory infections are tested for the cause of their acute viral infection, even among those who require hospitalization for moderate or severe illness [16]. Observed geographical differences, for example, could be more of a reflection of regional testing patterns rather than differences in virus activity. Molecular diagnostic tests that are user-friendly, point-of-care (POC), semi-automated, and low cost may facilitate further epidemiologic studies, particularly across resource-limited areas of the world [17].

In a study of participants recruited from a New York City tourist attraction, nasopharyngeal swabs, demographics, symptom surveys, medical history, and recent travel information were collected from 2,368 adults over two respiratory seasons [18]. Using a multiplex viral panel, a total of 6.2% of individuals tested positive for at least one virus, with 5.6% and 7.0% testing positive in the summer and winter arms, respectively [18]. Among those with viruses detected, 50.6% were human rhinovirus positive, 38.7% were coronavirus positive and 10.2% were positive for either adenovirus, metapneumovirus, influenza or parainfluenza [18]. Based on the surveys, the investigators concluded that up to 97% of the positive samples were collected from asymptomatic persons, although participants who tested positive were more likely to report symptoms than those who did not. The study concluded that there were high levels of asymptomatic respiratory virus shedding among this ambulatory population [18]. The accurate interpretation of diagnostic test results from multiplex assays performed on samples collected from symptomatic persons would certainly benefit from establishing similar baseline rates of pathogen detection in populations of healthy individuals. Baseline rates of pathogen detection, for example, may differ by age, setting, gender, or geography.

Several studies have quantified baseline pathogen rates directly or indirectly in the infant age group. In one study, infants receiving care for late-onset sepsis in neonatal intensive care units were evaluated for the frequency of respiratory viral infections using multiplex PCR [19]. Among the 135 cases with late onset bacterial sepsis, 6% also tested positive for a common respiratory virus (enterovirus, rhinovirus, coronavirus and/or parainfluenza virus [19]. Multivariate analysis revealed that the best predictor for testing positive for a respiratory virus was the caregiver’s clinical suspicion that the infant had an acute respiratory viral infection (*P* = 0.006; [19]). The investigators urged neonatologists to perform molecular testing for respiratory viruses as part of all sepsis evaluations with the goals of achieving more accurate/comprehensive test results, instituting appropriate infection control measures to prevent transmissions, and to de-escalate or discontinue the use of broad-spectrum intravenous antibiotics, as appropriate [19]. More recently, Bravo-Queipo-de-Llano et al. compared the incidence of acute respiratory tract infections in low birthweight infants admitted to their NICU before and after the start of the COVID-19 pandemic [20]. The investigators reported a substantial decrease in the number of ARIs following the onset of the pandemic and proposed that the results were due, at least in part, to widespread, nonpharmacologic interventions in place to prevent the spread of SARS-CoV-2 [20].

A year-long prospective surveillance study evaluated the frequency of respiratory pathogen detection in premature infants during their birth hospitalization [21]. Multiplex PCR capable of detecting 17 different respiratory viruses or virus subtypes was performed on nasal samples collected twice weekly until hospital discharge from study participants [21]. 52% of infants born at less than 33 weeks of gestational age tested positive for a respiratory virus at least once during their birth hospitalization [21]. Longer length of stay (70 vs. 35 days), prolonged ventilatory support (51 vs. 13 days), and twice the rate of bronchopulmonary dysplasia were associated with a positive versus a negative respiratory virus test [21]. This study documented frequent transmission of respiratory viruses to preterm infants during their birth hospitalization despite good adherence to infection control practices including frequent hand washing, routine use of gowns, gloves, and masks, and exclusion of non-parent visitors under 18 years-of-age. This study justified more intensive monitoring of respiratory infections via multiplex panels and encouraged further assessment of prevention and control strategies in this high-risk setting nearly a decade prior to the onset of the COVID-19 pandemic.

The likelihood for regional differences in respiratory infection epidemiology must also be considered. In 2018, a surveillance study was launched to evaluate the etiologies and seasonality of acute respiratory infections in Ecuadorian children less than 5 years of age seeking medical attention in an outpatient setting. Nasopharyngeal swabs were collected from 820 children and analyzed using multiplex PCR [22]. 80% of the samples were positive for the detection of at least one pathogen: rhinoviruses (44%), enteroviruses (17%), parainfluenza viruses (17%), RSV (15%), and influenza viruses (13%) [22]. When comparing results from samples collected across coastal versus highland regions, the onset and duration of annual seasonal RSV and influenza virus activity showed different patterns. Along the coast, RSV and influenza activity was highest between the rainy months from February to June, while virus activity in the high elevations, where temperate climate prevails year-round, was seen between November and March.

Among symptomatic children, co-detection of 2 or more pathogens in a single sample is common. In a 3-year prospective study of 201 children younger than 24 months who were hospitalized with a febrile respiratory illness, at least one respiratory virus was detected in 93% of the nasal samples collected while 28% tested positive for 2 or more viruses [22]. The investigators noted that there were no differences in illness severity between patients who tested positive for one versus more than one pathogen [22].

Annual seasonal outbreaks together with the emergence or re-emergence of novel respiratory viral pathogens create shifting and unpredictable public health problems. To limit the spread of new and known respiratory viral pathogens and, where necessary, to develop novel or modify existing immunizations and/or therapeutics, a circulating pathogen must be identified, isolated and characterized. Even when unavailable or not (yet) performed, the clinical presentation may offer clues as to the most likely culprit(s). When a definitive diagnosis may be necessary for a variety of reasons (e.g. outbreak, unusual cluster or disease manifestation, severe illness of uncertain etiology, or high-risk host), ultimately, the diagnosis is based on a combination of the clinical findings and any available laboratory evidence, including results of any virus- specific testing [23].

### Clinical and laboratory concepts for respiratory viral infection testing

Historically, virology diagnostic assays, such as virus isolation and culture, hemagglutination inhibition assays, enzyme immunoassays and direct fluorescent antibody staining were used. However, with better sensitivity, and technologic advances allowing workflow to be streamlined or automated, nucleic acid amplification tests (NAATs) are now considered the gold standard for respiratory virus diagnosis [24].

Viral isolation and culture testing is labor-intensive, typically requiring high-level technical training to perform and interpret, and requires the use of expensive biological reactants and/or laboratory equipment. Turnaround time to results can be as long as 14 days [25]. Care is needed with sample handling during transport from the bedside to the testing site both to minimize potential exposure to personnel and to maintain viability of virus. Biologic samples collected for virus culture should be stored on wet ice or under refrigeration until inoculated onto target cells. Many laboratories ceased performing respiratory viral cultures during the COVID-19 pandemic to minimize potential exposure to the pathogen during sample handling. Depending on the source and type of biologic sample being analyzed, higher level biosafety precautions may need to be implemented [25].

While rarely performed for general diagnostic purposes, propagating viruses in culture may be still be advantageous under certain circumstances. For example, the growth of certain viruses is required if formal antiviral susceptibility testing is necessary. Virus cultures may also be needed during outbreak investigations for optimal detection and characterization of the pathogen [25].

A rapid detection option for respiratory virus diagnostics are antigen-based tests such as lateral flow immunoassays [26]. Antigen POC tests are fast, relatively inexpensive, user-friendly, and can be performed outside the laboratory, some are even available for at-home testing [13]. Historically, in most contexts, antigen-based testing was associated with suboptimal sensitivity, specificity, and/or positive predictive value, each of which were highly dependent on the quality of the biologic sample being tested and the prevalence of virus circulating in the community at that time [26]. Also, unlike NAATs, antigen testing does not amplify the target signal, so a higher viral load is usually necessary for positive detection, leading to higher rates of false negative results [13]. 

### Multiplex molecular testing for ARI at Point-of-Care (POC)

There are several different types of molecular diagnostic tests, all of which are based on the amplification and detection of nucleic acids; DNA or RNA, from the pathogen(s) of interest. The number of FDA-approved molecular diagnostics for detection of respiratory pathogens has increased dramatically in recent years [27]. The number of targets included in these tests range from one to more than 20 pathogens including viruses and virus groups, and both typical and atypical bacterial targets. Specimen types deemed acceptable for testing also vary by assay including one or more of the following: nasal, nasopharyngeal or throat swabs, nasal aspirates, mid-turbinate swabs, and/or fluid collected by nasal wash or bronchoalveolar lavage [26]. Some molecular assays can be performed by staff with minimal laboratory training at POC or near-patient, while others must be run in centralized, certified laboratories by trained technologists.

The widespread availability of molecular diagnostic tests, many of which are multiplexed, has streamlined the ability to quickly identify the offending pathogen or pathogen group during the early phase of most respiratory tract infection outbreaks. Most of the newer molecular methods being used share the advantages of high sensitivity, high specificity, and same-day turn-around times when compared to older, traditional methods (Table 1 [28, 29]). Some POC test results can be available within minutes of sample collection [30]. For most of the available molecular assays, these advantages come at a significantly higher cost per test [28]. Reports are mixed regarding whether some or all of those costs can be recovered when rapid throughput and high levels of efficiency lead to changes in clinical management (e.g. use of or de-escalation of antibiotic therapy, decisions related to hospital admission or discharge).

**Table 1:** Summary of advantages and limitations of ARI diagnostic test methodologies

Diagnostic Test Methodology	Turnaround Time	Advantages	Limitations
Culture (viral or bacterial)	Days to weeks	• Specific • Drug susceptibility testing usually requires culturing the pathogen • The cultured agent can be stored in the laboratory and recovered if needed (e.g. drug susceptibility, outbreak investigation)	• Long turnaround time • High complexity • Low sensitivity for some sample types • Not all agents can be cultured • Handling biologic samples under culture conditions may present a biohazard
Antigen/antibody	15 min–3 h	• Rapid antigen tests have a turnaround time of 10–30 min • Some assays are CLIA-waived • Generally more affordable than other methods	• May have reduced sensitivity and specificity compared to culture or molecular testing
Molecular	15 min–3 h	• Increased sensitivity and specificity compared to culture and antigen/antibody methods • Rapid molecular tests allow for treatment (when available) at initial patient presentation • Some assays are CLIA-waived	• Many assays are moderate- to high- complexity • Non-rapid assays have a longer turnaround time • Generally more expensive than traditional tests • May not be reimbursed by payors • Do not differentiate between active infection and presence of residual pathogen specific nucleic acid
Multiplex molecular	15 min–3 h	• Increased sensitivity and specificity compared to culture and antigen/antibody methods • Tests for multiple pathogens with one sample • May reduce unnecessary antibiotic use • May reduce the need to perform other tests, such as chest radiographs • Able to detect the presence of 2 or more pathogens • Rapid tests allow for treatment, when available, during the patient encounter • Potential for customization using multiplex targets of interest (targeted treatment) • Often include pathogens that are challenging to diagnose using other methods • Smaller panels allow focused multiplex testing • Some assays are CLIA-waived	• Large panels may include unlikely or unnecessary targets based on the clinical scenario • Laboratory instruments needed to perform the assays are usually expensive. • The cost for test kits is typically commensurate with the number of targets present. Large panels may become cost prohibitive. • Do not differentiate between active infection and the presence of residual pathogen-specific nucleic acid • Co-detection of 2 or more targets may not indicate co-infection • Lack specificity for some groups of closely related viruses (e.g. rhinoviruses/enteroviruses) • In general, the sensitivity of multiplex assays are slightly lower than reported for monoplex assays with the same target

### Limitations of molecular diagnostic testing methods

Molecular assays make use of a variety of techniques, all of which rely on the detection of nucleic acids specific to a given pathogen. Due to the persistent nature of nucleic acids after cell death and their persistence in the environment, amplicon contamination resulting in false positives is a concern with molecular tests. These false positives can be mitigated by the use of internal controls inherent in the assays. Biological false positives in asymptomatic individuals could represent pre-symptomatic infections or residual presence of nucleic acid after the pathogen itself is no longer replication sufficient. Available molecular diagnostic tests used for the detection of common respiratory pathogens are unable to distinguish between the presence of nonviable agents of infection, active infection with replication competent pathogens, asymptomatic carriage or shedding, or quantitate levels of contagion. Chemical inhibition of PCR amplifying enzymes can result in false negatives. False negatives are more difficult to measure but are thought to be relatively low due to the high sensitivity and specificity of nucleic acid tests (> 95%; Table 2 [31–66]) and the use of positive and negative controls in each PCR run. Commercially available molecular panels are unable to distinguish between certain related viruses. For example, due to high rates of genetic similarity between human rhinoviruses and human enteroviruses, current multiplex platforms cannot reliably differentiate them [67, 68]. As such, a positive diagnostic test result reports detection of human rhinovirus/enterovirus. Such a result may be interpreted as the detection of a human rhinovirus, a human enterovirus, or both. Further characterization of the virus(es) present in the biological sample requires testing beyond the assays typically performed in a clinical diagnostic laboratory, such as partial or complete sequencing of the viral genome(s).

**Table 2:** Sensitivity, specificity, and complexity of ARI diagnostic test methodologies

Diagnostic Test Methodology	Sensitivity Range*	Specificity Range*	One or more CLIA-Waived Assays available
Culture	42–92%	90–99%	No
Direct fluorescent assay (DFA)	62–100%	99–100%	No
Immunofluorescent assay (IFA)	59–85%	90–99%	No
Rapid antigen detection test (RADT)	24–95%	90–95%	Yes
Nucleic acid amplification test (NAAT)	80–100%	82–100%	Yes
Multiplex molecular assay (high complexity)	80–100%	82–100%	No
Multiplex molecular assay (low complexity)	87–95%	95–100%	Yes

*Sensitivity and specificity vary by pathogen and by platform

Regardless of the type of test performed, personnel involved with POC testing should be trained on basic infection control practices. While many of these tests are Clinical Laboratory Improvement Amendments (CLIA)-waived for POC, they are still complex tests and require training, proper positive and negatives controls, and continuous monitoring for reliability. Newer tests make use of kits and cartridges which are user-friendly and reduce human error and environmental contamination. Validation studies in real world settings should be conducted and compared to manufacturer’s stated expectations.

### Knowledge gaps with asymptomatic carriage and Co-Infections

Several studies have questioned the validity and utility of multiplex panels. Additionally, there are gaps in clinical knowledge. To make effective decisions, clinicians must understand the significance of the results obtained from these panels. Most large-scale results involve data largely derived from samples collected from symptomatic patients [18]. The pathogen detected is therefore presumed to be the cause of the patient’s symptoms. The presumption is largely correct, but given the nuances of molecular testing described above, in some cases, the detected agent(s) may represent residual nucleic acid from a prior infection or asymptomatic carriage/shedding [44, 45]. Additionally, the patient’s symptoms may be caused by a different pathogen not represented in the assay.

A study was performed in children under treatment for malignancy who were hospitalized with fever and neutropenia [69]. In addition to the diagnostic testing ordered by the admitting physician, a nasopharyngeal swab was collected for testing using a multiplex PCR-based respiratory panel. Respiratory symptoms were absent in most of the children enrolled, yet 52% tested positive for one or more respiratory viral pathogens [69]. In contrast, the diagnostic evaluation ordered by the admitting physician revealed a microbiologic cause for the fever illness in 18% of the children enrolled [69]. While use of multiplex molecular panels allows for co-detection of 2 or more pathogens from the same biologic sample, the clinical significance of pathogen co-detection, if any, remains unknown [28, 70–73].

Finally, the detection of nucleic acids from 2 or more pathogens from the results of a single multiplex panel may be analytically true but difficult for the clinician to correctly interpret. One commercial assay reports the ability to quantify bacterial targets and has proposed a threshold above which an organism likely represents a viable pathogen [74]. The use of validated cut-off points which differentiate between non-viable and infectious pathogens may one day allow clinicians to confirm the presence of infection and/or the presence of a replication-competent pathogen. However, the validity of such an interpretation has yet to be confirmed in real-world clinical studies. More data are needed to guide and inform best practices for optimizing the use of syndromic multiplex testing panels when evaluating children with suspected respiratory viral infections [28, 75].

### Multiplex molecular assays for screening

Multiplex molecular panels range in size from only two pathogens to large infectious disease syndromic panels with potentially over a hundred targets. While larger panels may have a role in screening for pandemic, epidemic, and unidentified pathogen outbreaks, many smaller panels, or platforms with the ability to select specific pathogens of interest, may be more clinically useful and cost-effective in healthy children and outpatient populations. Molecular and multiplex testing may in some cases be more costly, but the high throughput, excellent sensitivity and specificity, and rapid turn-around times which are features of these assays are useful for screening and diagnosing patients presenting with acute respiratory symptoms. Multiplex panel screening may reduce nosocomial infections and improve isolation practices.The impact of using multiplex respiratory panel diagnostics on care and costs for high-risk children presenting to outpatient settings remains unclear. Randomized controlled trials to evaluate the clinical impact of using available multiplex diagnostics as a standard approach in the evaluation of acute illnesses in individuals with pre-existing immunocompromising conditions, underlying chronic respiratory or cardiac conditions, or other comorbidities have not been published, but should be considered.

### Clinical utility of multiplex molecular tests

Studies evaluating the clinical utility of rapid POC molecular tests for ARIs have provided variable results – from beneficial to no apparent benefit, depending upon the parameters measured and the populations under examination. The clinical utility of these tests for diagnosis of ARIs varies according to the implementation strategies for the diagnostic test, the setting in which the biologic samples are collected (e.g., inpatient, outpatient), characteristics of the target population being tested, (e.g. age, pregnant, general health, immunocompromised), illness severity under evaluation, and characteristics of the molecular test being used (e.g., number and type of targets tested, turn-around-time, assay sensitivity and specificity). For example, the clinical utility of a POC molecular test performed on patients in emergency departments (ED) or urgent care centers will be deemed more favorable if the turn-around-time is rapid, ideally before the patient has left the clinic [28].

Other rapid tests such as antigen immunoassays and viral direct fluorescent assay (DFA) diagnostic tests used prior to the adoption of multiplex molecular tests have shown benefits for certain patient outcomes. In one study, patients who tested positive for influenza using a rapid antigen immunoassay were less likely to be prescribed antibiotics, had fewer additional diagnostic tests performed, were more likely to receive antiviral therapy, and were less likely to be hospitalized than patients with negative test results [76]. Even when the less sensitive respiratory virus DFA diagnostic tests are performed, if results were available within 24 h, their routine use was associated with substantial reductions in the duration of hospitalization, fewer prescriptions for antibiotic treatment, fewer requests for other microbiological diagnostic tests [77]. A meta-analysis of selected studies performed in children presenting to the emergency department with signs and symptoms consistent with ARIs showed that the use of rapid viral diagnostic tests was associated with lower rates of chest radiographs (RR 0.77, 95% CI 0.65 to 0.91) [69]. There was a trend toward decreased antibiotic use in the ED, but it was not statistically significant [69]. Rapid viral testing had no effect on length of ED visits, blood or urine testing [69, 78–80].

Results from observational case-controlled studies performed in hospitalized patients highlight several advantages associated with molecular testing, especially for assays with a rapid turn-around time. Positive results from multiplex PCR-based rapid respiratory virus diagnostic testing have been shown to be associated with less antibiotic use in hospitalized children and adults [81–83].

Several small, single-center, observational studies were conducted to evaluate the clinical utility of multiplex syndromic respiratory panel diagnostic testing in hospitalized children presenting with symptoms consistent with acute respiratory infection. These studies have shown clear reductions in inappropriate antibiotic use, increases in appropriate antiviral use [84], and fewer orders for ancillary laboratory and radiographic testing [28, 83, 85, 86]. However, another similarly designed study failed to demonstrate any clear favorable clinical utility in routinely implementing multiplex diagnostic testing [87]. In contrast, results from studies on the clinical utility of multiplex diagnostic testing among hospitalized pediatric patients, including preterm neonates, have been favorable [19–21].

Due to mixed results concerning clinical utility, more studies are needed to determine the appropriate use of these multiplex molecular tests or NAATs in different settings and among different patient populations. Based on existing evidence, the Association for Diagnostics and Laboratory Medicine (ADLM) has published a guidance document for the laboratory diagnosis of respiratory viruses [26]. For the diagnosis of pediatric patients with respiratory symptoms, they recommend limiting multiplex respiratory pathogen panels to hospitalized children, immunocompromised patients, or children with underlying conditions [26]. They caution that small targeted, panels focusing on seasonal outbreaks such as influenza and RSV may be sufficient [26]. The American Academy of Pediatrics (AAP) recommends SARS-CoV-2 testing for any children who have symptoms of COVID-19 or have had contact with a confirmed case of the virus.

### Cost effectiveness studies

Systematic cost effectiveness analyses on the routine use of these molecular multiplex diagnostic tests in different age groups, in patients with and without underlying chronic medical conditions, or across different health care settings, have not been evaluated [28]. One model that evaluated the economics of each of the available rapid influenza diagnostic test types in a pediatric ED setting, found that the use of rapid multiplex PCR-based testing was more cost effective than conventional influenza NAAT analysis, DFA testing, and rapid antigen testing [77, 88]. Similarly, a prospective study designed to evaluate the economic impact of a rapid multiplex PCR-based influenza A/B assay on provider decision-making showed the approach had potential to be associated with substantial cost savings and improved antiviral use [75, 77]. Taken together, these studies support the clinical utility and cost-effectiveness of using rapid, POC testing, particularly for influenza and influenza-like illnesses.

In a prospective study assessing the impact of reverse-transcriptase PCR (RT-qPCR) influenza testing (98.8% sensitivity; 98.5% specificity) on physician decision-making in the ED, they analyzed changes in admission/discharge status, medical procedures, antiviral and antibiotic prescribing, and laboratory studies [70]. They found that an influenza diagnosis by RT-qPCR altered patient management in 61% of the cases. This resulted in a cost savings of between $42,270 to $49,420 over 143 patients or a projected cost savings of approximately $200 per ED visit [70]. They projected that over 2,000 ED patient visits a cost savings of greater than $578,000 due to deferred admissions and reduction in antiviral prescribing [70]. Another clinical trial studied the clinical utility of knowing results of a 17-plex, PCR-based respiratory pathogen panel with a rapid turn-around time. The authors of the study found no statistically significant differences in hospitalizations, length of hospital stay, or use of antibiotics even when test results were available within 12 to 36 h of sample collection. A formal cost analysis was not included in the report, but the lack of significant differences between the study arms suggests that the routine use of rapid multiplex respiratory pathogen diagnostics in children presenting to the pediatric ED cannot be assumed to be cost saving in all outpatient settings [83, 84].

Cost-effectiveness evaluations of these molecular diagnostics should consider potential benefits in terms of reducing antimicrobial resistance (AMR). If detection of viral pathogens reduces unnecessary antibiotic use for viral ARIs, this could lead to less selection of resistant bacteria and benefits at the population level. It is difficult to quantify the potential averted costs of reducing AMR, but if multifaceted actions are not taken to reduce emergence and spread of AMR, the World Health Organization and World Bank estimate that AMR could result in US$ 1 trillion additional healthcare costs by 2050, and US$ 1 trillion to US$ 3.4 trillion gross domestic product (GDP) losses per year by 2030 [89].

### Insurance coverage

The U.S. Medicare Coverage Database (MCD) outlines the following covered indications:

Respiratory pathogen panel testing in the outpatient setting by a Part B provider (e.g., physician’s office, independent clinical laboratory) will be considered medically reasonable and necessary when panels with 5 or fewer respiratory pathogens are performed, the outpatient setting is equipped to deliver timely reporting of results to providers, and for patients where the test result aids clinical management with the goal of an improved health outcome for the patient [90].

The MCD considers panels with greater than 5 respiratory pathogens performed in the Part B outpatient setting as not medically reasonable and necessary due to a lack of supporting data in the peer-reviewed literature [90].

The American Thoracic Society and Infectious Diseases Society of America’s (IDSA) clinical practice guidelines addresses high priority questions using systematic reviews of high-quality studies. It is noted that the evidence base was often insufficient and emphasizes the continued importance of clinical judgment and experience, and the great need for research [90].

However, the MCD makes exceptions. For example, it follows ISDA guidelines for use of multiplex RT-PCR assays targeting a panel of respiratory pathogens, including influenza viruses, in hospitalized immunocompromised patients [90]. All, or nearly all payers, reimburse for the use of these tests in immunocompromised patients and for those who have severe respiratory illness requiring hospitalization. Providers and patients outside the U.S. are frequently faced with additional limitations and variations on reimbursement. In the EU, member states can have individual regulations that do not facilitate directives from pan-European legislation [91–93]. Likewise other countries such as Canada and Asian, African, and Latin American countries may lack or have distinct reimbursement regulations [94–96]. Most countries will reimburse for specific use cases involving immunocompromised patients or those with severe illness, though other reimbursement efforts can differ country to country depending on whether insurance is a private or public offering. When clinicians are aware of reimbursement limitations, the current payer decisions have the potential to influence clinician behaviors, discourage the use of multiplex testing even when deemed clinically necessary by the clinician, and restrict test availability and/or the ability to use these important diagnostic tools in many institutions. When clinicians are unaware of payer decisions, they may unintentionally push higher costs to the patient who must cover these costs out-of-pocket. In cases of mild ARI, some argue that molecular diagnostics may not provide actionable information for clinicians, may not be superior to clinical judgment alone, and in fact may be contributing to excess healthcare costs [82, 97]. Nevertheless, the impact of these payer decisions on clinical outcomes, patient medical costs and debts, and potential worsening of existing health disparities should be investigated.

### Public awareness and COVID-19 influence

In the United States, all testing of human biologic samples for the purposes of diagnosis, prevention, or treatment of disease is regulated by the Food and Drug Administration under the Clinical Laboratory Improvement Amendments of 1988 (CLIA). Diagnostic tests that meet precise definitions for low risk, low error, and low complexity may be issued a CLIA certificate of waiver allowing them to be used in settings other than certified clinical laboratories. Point of care tests that are approved by the FDA for home use automatically qualify for a CLIA waiver. SARS-CoV-2- specific assays were the first over the counter (OTC), CLIA-waived respiratory virus diagnostic tests to become available to the public [98].

While payers generally discourage the use of respiratory viral diagnostics in outpatient settings and for most otherwise healthy patients, their increasingly widespread commercial availability allow individuals to access testing without seeking care from a medical provider. Terms and concepts that were once only well understood by those with strong healthcare backgrounds, such as monoclonal antibodies, emergency use authorization, POC testing, and PCR, have come to the attention of the general population. More than 30 CLIA-waived respiratory virus diagnostic assays targeting a wide variety of respiratory pathogens are now available [98]. The recent experience of near-universal public access to government subsidized, over the counter, rapid testing kits for SARS-CoV-2, may serve to raise public awareness regarding the similar availability of diagnostic tests for other pathogens. Data regarding public perceptions of the conveniences of rapid test results, collecting their own respiratory samples, then performing and interpreting OTC tests at home are largely unknown.

As public awareness and availability of OTC molecular assays for the detection of an expanding array of respiratory pathogens increases, new challenges may present themselves. The interpretation of molecular results is complicated and requires a detailed understanding of how the test works, its positive predictive value, and the sensitivity and specificity of each target being tested. Correct interpretation of the assay results are also essential for determining whether any additional medical intervention(s) should be considered. Clinicians already face a similar dilemma when attempting to balance stewardship of healthcare resources and antibiotic prescribing practices with subsets of patients who expect to receive an antibiotic prescription based on their own preference or bias. For example, following telemedicine encounters for ARIs in their children, parents reported increased satisfaction with the visit when a prescription for antibiotics was provided [92, 99]. To counter this trend in patient expectations, health care systems and clinical laboratories should be proactive by developing respiratory virus testing algorithms that best support clinicians and optimize patient care [75].

### Updating present guidelines

Presently, IDSA considers respiratory NAATs as most useful in intermediate disease severity and high pretest probability– where a positive test can result in therapy focused on that particular pathogen and a negative test avoids unnecessary antibiotic administration [28]. Timely updates of NAAT use warrants consideration as more data on clinical outcomes, costs and labor analyses are collected and compared to historical alternatives. Decision trees based on patient-centered vignettes can be created and used to determine the best test options, be they panels with a few viral or bacterial targets, more extensive multiplex panels, platforms that allow specific pathogen selection from larger panels, or no testing at all. As new pathogens emerge or old ones rebound, the use of various molecular methods must be reevaluated based on emerging trends and epidemiological data. Ongoing surveillance studies on symptomatic, asymptomatic, healthy, and high-risk individuals will be needed to optimize panels and interpret their results. Movement in “seasonality” of respiratory pathogens due to climate change and global travel may drive the evaluation of year-round multiplex panel testing for purposes of screening, surveillance, and pretest probability to prevent and address future outbreaks, epidemics, and pandemics.

### Future considerations

Many studies support the clinical utility and cost-effectiveness of performing multiplex respiratory virus testing in outpatient and inpatient settings. With that said, not all reports strongly endorse routine use for all patients across all settings due to the associated costs of the assays and uncertainty regarding their utility across certain patient populations and/or clinical settings. Going forward, institution-specific and/or laboratory-specific studies can help guide decisions regarding implementation and/or ongoing use of such assays. Diagnostic stewardship decisions related to use of multiplex respiratory pathogen assays, which ones to offer, and to whom, require engagement and discussion with stakeholders across a number of disciplines. Bringing together clinical microbiology and virology directors, clinicians across a variety of specialties, hospital administrators, and others is essential to ensure that high quality, cost-effective respiratory virus testing algorithms are available for the purpose of optimizing clinical care. While many institutions and clinical laboratories are reluctant to offer the current, most comprehensive rapid multiplex respiratory pathogen PCR-based diagnostics for ‘all patients, all sites, all circumstances’, best -practice diagnostic stewardship decisions can be developed using parameters such as patient age, underlying risk factors, location of the encounter, sensitivity and specificity of multiplex assays being considered, and time to results. Awareness of, and links to local, regional, and statewide respiratory virus surveillance may also help to inform stewardship decisions based on seasonal activity of specific pathogens and current outbreaks, epidemics, or pandemics. As new and more sophisticated laboratory diagnostic tools become available, the importance of establishing formal, multidisciplinary stewardship teams responsible for the development and maintenance of clinically useful, cost-effective approaches to laboratory diagnostics grows.

## Conclusions

Due to the significant increase in the number of FDA-cleared, CLIA-waived, and CLIA moderate-complexity molecular and multiplex tests in the past few years, clinicians and microbiology laboratories now have more options which generate results within minutes or hours. Rapid molecular single- or multiplex testing has the potential to improve timely and accurate diagnosis, reduce inappropriate antibiotic use, decrease reliance on chest radiographs, increase timely antiviral administration, reduce the length of stay, reduce the number of clinical visits, and, ultimately, improve patient outcomes. Accurate and timely diagnosis and treatment of ARIs can prevent progression to more serious pathologies, especially in the young, elderly, immunocompromised, and other high-risk groups. Using judicious and thoughtful screening protocols, these tests may also assist in the implementation of agent-specific infection controls and isolation precautions in clinical and community settings. Regional and global disease surveillance may be enhanced with the implementation of user-friendly multiplex molecular panels by contributing data on the presence of asymptomatic carriers and/or co-infections and can fill in the gaps which exist in our understanding of the epidemiology of respiratory infections.

## Data Availability

No datasets were generated or analysed during the current study.

## Declarations

### Manuscript Preparation

Medavera provided assistance with preparing and editing the manuscript.
