# Correction: Rapid diagnosis of acute pediatric respiratory infections with Point-of-Care and multiplex molecular testing

**DOI:** 10.1007/s15010-025-02593-x

**Published:** 2025-06-30

**Authors:** Jane M. Caldwell, Claudia Mily Espinosa, Ritu Banerjee, Joseph B. Domachowske

**Affiliations:** 1 Medavera, Inc., Springfield, MO 65807 USA; 2 Division of Pediatric Infectious Diseases, University of South Florida, Tampa, FL USA; 3 Division of Pediatric Infectious Diseases, Vanderbilt University Medical Center, Nashville, TN USA; 4 Upstate Medical University, Syracuse, NY USA

**Correction: Infection**

10.1007/s15010-025-02553-5

The article “Rapid diagnosis of acute pediatric respiratory infections with Point-of-Care and multiplex molecular testing”, written by Jane M. Caldwell · Claudia Mily Espinosa · Ritu Banerjee · Joseph B. Domachowske, was originally published under exclusive license to Springer-Verlag GmbH Germany, part of Springer Nature. As a result of the subsequent decision to publish the article under the open access model, the article’s copyright notice was changed on 11 June 2025 to © The Author(s) 2025 and the article is now distributed under a Creative Commons Attribution

Open Access This article is licensed under a Creative Commons Attribution 4.0 International License, which permits use, sharing, adaptation, distribution and reproduction in any medium or format, as long as you give appropriate credit to the original author(s) and the source, provide a link to the Creative Commons licence, and indicate if changes were made.

The images or other third party material in this article are included in the article’s Creative Commons licence, unless indicated otherwise in a credit line to the material. If material is not included in the article’s Creative Commons licence and your intended use is not permitted by statutory regulation or exceeds the permitted use, you will need to obtain permission directly from the copyright holder.

To view a copy of this licence, visit http://creativecommons.org/licenses/by/4.0/.

